# Efficient and Simple Synthesis of 6-Aryl-1,4-dimethyl-9*H*-carbazoles

**DOI:** 10.3390/molecules13061312

**Published:** 2008-06-11

**Authors:** Anna Caruso, Anne Sophie Voisin-Chiret, Jean-Charles Lancelot, Maria Stefania Sinicropi, Antonio Garofalo, Sylvain Rault

**Affiliations:** 1 Université de Caen Basse-Normandie, U.F.R. des Sciences Pharmaceutiques, Centre d’Etudes et de Recherche sur le Médicament de Normandie, UPRES EA-4258 FR CNRS INC3M, 5, rue Vaubénard, 14032 Caen Cedex, France; 2 Università della Calabria, Dipartimento di Scienze Farmaceutiche, 87036 Arcavacata di Rende (CS), Italy

**Keywords:** Carbazole, ellipticine, boronic acid, cross-coupling reaction

## Abstract

A synthetic method for the preparation of 6**-**aryl-1,4-dimethyl-9*H*-carbazoles involving a palladium catalyzed coupling reaction of 1,4-dimethyl-9*H*-carbazole-6-boronic acids and (hetero)aryl halides is described.

## Introduction

Several alkaloids based on a carbazole structure are known to possess interesting biological activities. Antitumor, antibacterial, anti-inflammatory, psychotropic and anti-histamine properties have been attributed to many of such compounds [1]. More recently, carbazoles have also been used as photoconductors, semiconductors and for their light-emitting properties, making them interesting organic tools for physics experiments [2]. The introduction of functional groups onto the carbazole scaffold is essential to generate compounds suitable for biological and physical investigations. Standard electrophilic substitution reactions on readily available 1,4-dimethyl-9*H*-carbazoles are usually unselective, taking place indifferently at positions 3, 6, or 9 [3,4], while methods for the selective substitution on a given position are lacking. We herein describe a method for the conversion of (5,8-dimethyl-9*H*-carbazol-3-yl)boronic acid, easily prepared starting from the known 6-bromo-1,4-dimethyl-9*H*-carbazole, to the corresponding 6-aryl-1,4-dimethyl-9*H*-carbazoles by a Suzuki-Miyaura cross-coupling reaction.

**Figure 1:**
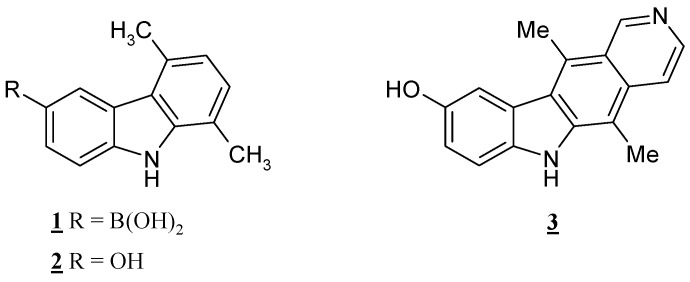
Carbazol-3-yl boronic acid **1**, its 3-hydroxy derivative **2** and hydroxyellipticine (**3**).

Many examples of Suzuki-Miyaura cross-coupling reactions between heterocyclic halides and phenyl boronic acids have been so far described [5,6], while the use of heteroaryl boronic acids or esters in the type of reactions is of limited utility [7,8]. Nevertheless, the reactivity of heteroaryl boronic derivatives continues to be of great interest. We reported in a previous paper the synthesis of carbazol-3-yl-boronic acid **1** (Figure 1), which was converted, via a hydroxydeboronation reaction, into the corresponding 3-hydroxy derivative **2** [9], a precursor of new analogs of the anticancer agent 9-hydroxyellipticine (**3**) [10,11,12].

Herein, we describe an efficient method involving a cross-coupling reaction, which would allow the preparation of a library of 6-aryl substituted carbazoles, as potential antitumoral agents. The key step is the reaction of (5,8-dimethyl-9*H*-carbazole-3-yl)boronic acid with (hetero)aryl halides to give 3-(hetero)arylcarbazole derivatives. Some typically used reaction conditions proved to be ineffective when applied to 6-bromo-1,4-dimethyl-9*H*-carbazole and (hetero)arylboronic acids. 

## Results and Discussion

**Scheme 1:**
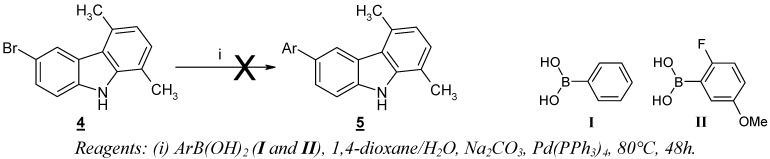
Cross-coupling reaction with 6-bromo-1,4-dimethyl-9*H*-carbazole **4**.

The starting 6-bromo-1,4-dimethyl-9*H*-carbazole (**4**) was prepared by a known procedure [13,14]. Classic cross-coupling conditions (Na_2_CO_3_/Pd(PPh_3_)_4_/1,4-dioxane/water/80°C) [6] applied to the reaction of this compound with arylboronic acids **I** and **II** proved to be ineffective for the production of the desired 6-aryl-1,4-dimethyl-9*H*-carbazoles **5** (Scheme 1).

*Reagents: (i) ArB(OH)_2 _(**I** and **II**), 1,4-dioxane/* *H_2_O, Na_2_CO_3_, Pd(PPh_3_)_4_, 80°C* *, 48h.*

**Scheme 2:**
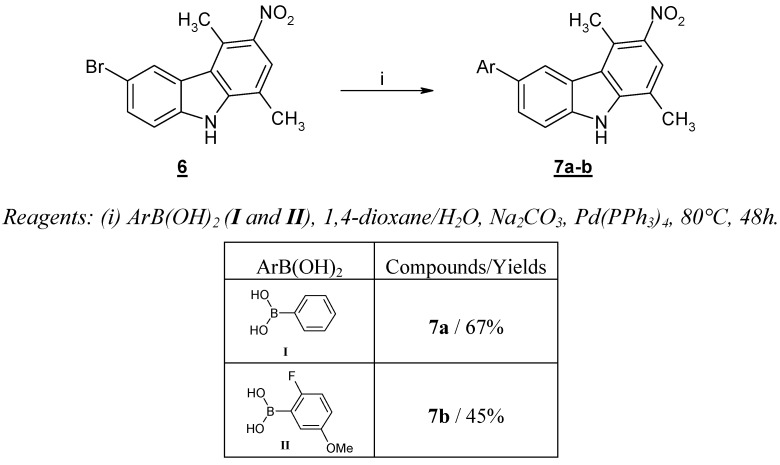
Cross-coupling reaction with 6-bromo-1,4-dimethyl-3-nitro-9*H*-carbazole (**6**).

However a similar experimental procedure was effective when applied to the reaction of arylboronic acids **I** and **II** and 6-bromo-1,4-dimethyl-3-nitro-9*H*-carbazole (**6**), giving aryl-1,4-dimethyl-3-nitro-9*H*-carbazoles **7a **and **7b**, in reasonable yields (Scheme 2). As already described in numerous cases, the presence of a strong electron-withdrawing group favored the success of the reaction [15].

Faced with these difficulties and also because the nitro group was considered undesirable, we have studied the reverse strategy, consisting in the preparation of the boronic acid **1**. We have already described the synthesis of this *N*-unprotected boronic acid **1** [9] starting from 6-bromo-1,4-dimethyl-9*H*-carbazole (**4**). 

**Scheme 3:**
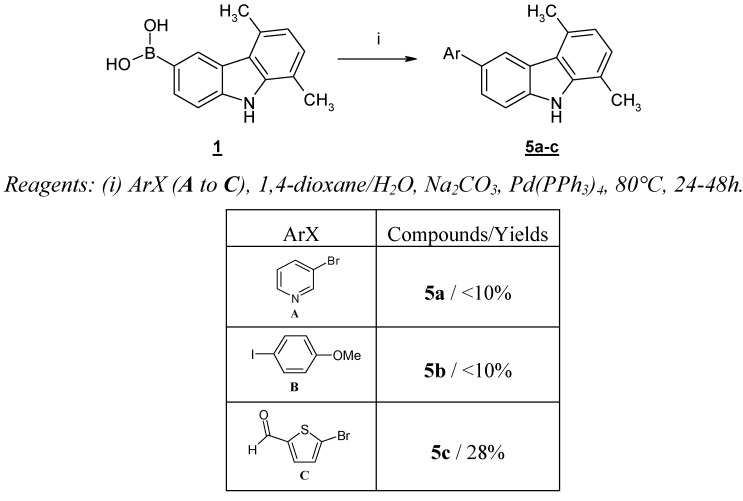
Cross-coupling reaction with 5,8-dimethyl-9*H*-carbazole-3-boronic acid (**1**).

Unfortunately, this boronic acid is a very poor partner in cross-coupling reactions and yields obtained with (hetero)aryl halides such as 3-bromopyridine (**A**) and *p*-iodoanisole (**B**) remained very modest (<10%), the exception being a heteroaryl halide bearing an electron withdrawing group such as 5-bromo-2-thiophenecarboxaldehyde (**C**), which gave the product **5c** in 28% yield (Scheme 3).

These failures prompted us to protect the nitrogen atom of 5,8-dimethyl-9*H*-carbazole-3-boronic acid (**1**). Thus, we prepared *N*-Boc-5,8-dimethyl-9*H*-carbazol-3-boronic acid (**8**), which was obtained after *N*-Boc-protection and successive lithiation-boronation of compound **4**. We previously described the use of this reaction sequence for the preparation of 5,8-dimethyl-9*H*-carbazole-3-ol [9].

**Scheme 4:**
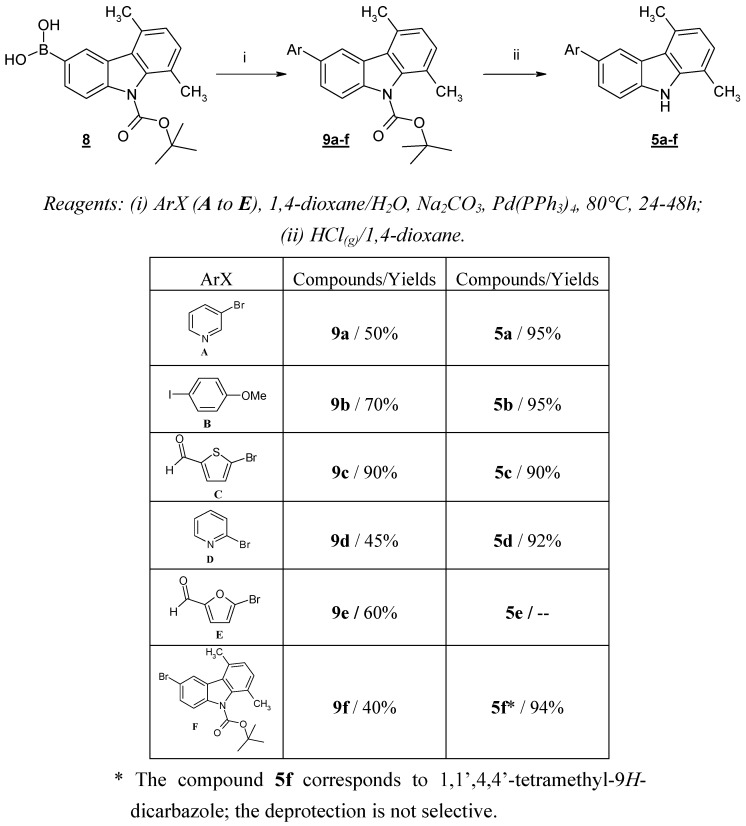
Cross-coupling reaction with *N*-Boc-5,8-dimethyl-9*H*-carbazol-3-boronic acid (**8**).

Compound **8** was then subjected to the Suzuki cross-coupling reaction with a variety of (hetero)aryl halides under standard conditions [6]. An aqueous solution of Na_2_CO_3_ and Pd(PPh_3_)_4_ was slowly added to a mixture of compound **8** and the appropriate (hetero)aryl halide in 1,4-dioxane at room temperature. The resulting mixture was then heated to reflux for the given time. After the usual work-up, compounds **9a-f** were obtained in good yields. The *N*-deprotection of **9a**-**f** was carried out with gaseous HCl in 1,4-dioxane, giving compounds **5a**, **5b**, **5c**, **5d **and **5f** in quantitative yields (Scheme 4). We were unable to isolate compound **5e**, probably due to the instability of the furfural moiety in an acidic medium.

## Conclusions

**Figure 2:**
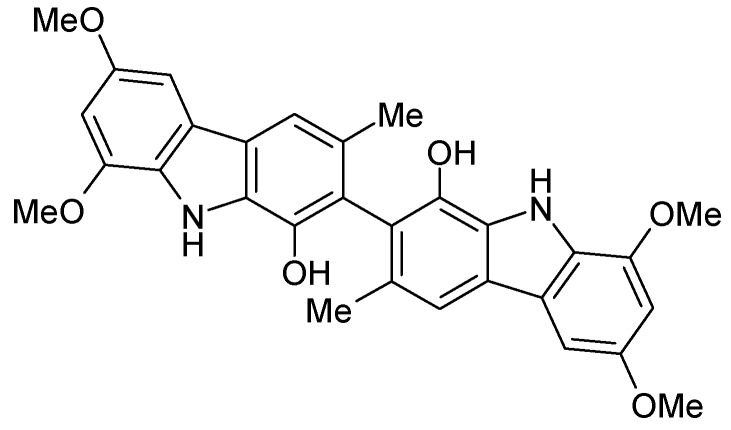
Clausenamine A.

We have found a good methodology to produce 6-aryl-1,4-dimethyl-9*H*-carbazoles using a *N*-protected 5,8-dimethyl-9*H*-carbazol-3-boronic acid. This methodology allows us to prepare new derivatives with potent interest in cancerology, especially biscarbazoles similar to Clausenamine A (Figure 2) [16,17,18,19].

## Experimental

### General

Commercial reagents were purchased from Aldrich, Acros Organics or Alfa Aesar and used without additional purification. Melting points were determined on a Kofler melting point apparatus. IR spectra were taken with a Perkin Elmer BX FT-IR. Mass spectra were taken on a JEOL JMS GCMate spectrometer at ionizing potential of 70 eV (EI) or were performed using a LC-MS Waters Alliance 2695 spectrometer (ESI+). ^1^H-NMR (400 MHz) was recorded on a JEOL Lambda 400 spectrometer; ^13^C-NMR were not recorded due to the low solubility of the compounds. Chemical shifts are expressed in parts per million downfield from tetramethylsilane as an internal standard. Thin layer chromatography (TLC) was performed on silica gel 60F-264 (Merck). 

### General procedure for the preparation of compounds **7a-b**

The preparation of 1,4-dimethyl-3-nitro-6-phenyl-9*H*-carbazole (**7a**) is reported as a representative example: to a solution of 6-bromo-1,4-dimethyl-3-nitro-9*H*-carbazole (**6**, 1.50 g, 4.70 mmol) in 1,4-dioxane (70 mL), kept under an argon atmosphere, phenylboronic acid (**I**, 0.63 g, 5.17 mmol), Na_2_CO_3_ (1.25 g, 11.79 mmol) in H_2_O (3.50 mL) and Pd(PPh_3_)_4 _(0.30 g, 0.26 mmol) were added sequentially. The reaction mixture was heated to reflux for 48h, and then volatiles were evaporated under reduced pressure. The solid formed was recrystallized from acetonitrile to give *1,4-dimethyl-3-nitro-6-phenyl-9H-carbazole* (**7a**). Yellow solid (67 % yield), mp = 226 °C; IR (KBr): 3451, 1584, 1521, 1302, 1301, 817, 758 cm^-1^; ^1^H-NMR (DMSO-*d*_6_) δ 11.94 (s, 1H, NH); 8.35 (s, 1H, H2); 7.86 (s, 1H, H5); 7.83-7.83 (m, 7H, H7, H8, C_6_H_5_); 2.96 (s, 3H, CH_3_); 2.55 (s, 3H, CH_3_); MS (EI) *m/z* (%): 316 (M^+ •^, 98), 271 (48) (M^+ •^-NO_2_). 

### 1,4-Dimethyl-3-nitro-6-(2’-fluoro-5'-methoxyphenyl)-9H-carbazole (**7b**)

Prepared following the general procedure but employing 2-fluoro-5-methoxyphenylboronic acid (**II**) in place of **I**. Yellow solid (45 % yield), mp = 240 °C; IR (KBr): 3309, 1578, 1299, 1205, 1034, 810, 756 cm^-1^; ^1^H-NMR (DMSO-*d*_6_) δ 12.06 (s, 1H, NH); 8.36 (s, 1H, H2); 7.87 (s, 1H, H5); 7.71-7.65 (m, 2H, H8, H6’); 7.28-7.23 (m, 1H, H7); 7.17-7.14 (m, 1H, H4’); 6.95-6.93 (m, 1H, H3’); 3.80 (s, 3H, OCH_3_); 2.99 (s, 3H, CH_3_); 2.58 (s, 3H, CH_3_); MS (ESI^+^): 364 (M^+^+1).

### General procedure for the preparation of compounds **9a-f**

The preparation of 1,4-dimethyl-6-pyridin-3-yl-9-*tert*-butoxycarbonyl-9*H*-carbazole (**9a**) is reported as a representative example: to a solution of 3-bromopyridine (**A**, 0.08 mL, 0.80 mmol) in 1,4-dioxane (50 mL), kept under an argon atmosphere, 9-*tert*-butoxycarbonyl-5,8-dimethyl-9*H*-carbazole-3-boronic acid (**8**, 0.30 g, 0.88 mmol), Na_2_CO_3_ (0.21 g, 2 mmol) in H_2_O (2 mL) and Pd(PPh_3_)_4 _(0.05 g, 0.04 mmol) were added sequentially. The reaction mixture was heated to reflux for 48 h, and then concentrated under reduced pressure. The solid residue was recrystallized from acetonitrile. Compound **9a** was obtained as a light brown solid (50 % yield), mp = 167 °C; IR (KBr): 3435, 2972, 1731, 1458, 1288, 1246, 1145, 1084, 809, 721, 619 cm^-1^; ^1^H-NMR (DMSO-*d*_6_) δ 9.01 (s, 1H, H2’); 8.60-8.56 (m, 1H, H6’); 8.35 (s, 1H, H5); 8.20 (d, *J*_H4’-5’_ = 7.8 Hz, 1H, H4’); 8.11 (d, *J*_H7-8 _= 8.8 Hz, 1H, H7); 7.87 (d, *J*_H8-7 _= 8.8 Hz, 1H, H8); 7.53-7.50 (m, 1H, H5’); 7.24 (d, *J*_H2-3_ = 7.8 Hz, 1H, H2); 7.15 (d, *J*_H3-2_ = 7.8 Hz, 1H, H3); 2.84 (s, 3H, CH_3_); 2.40 (s, 3H, CH_3_); 1.67 (s, 9H, 3CH_3_); MS (EI) *m/z* (%): 372 (M^+ •^, 1), 316 (100) (M^+ •^- *t*Bu), 272 (100) (M^+ •^- CO_2_*t*Bu). MS (ESI^+^): 373 (M^+^+1).

### 6-(4’-Methoxy-phenyl)-1,4-dimethyl-9-tert-butoxycarbonyl-9H-carbazole (**9b**)

Following a procedure identical to that described for **9a**, but using 4-iodoanisole (**B**, 0.30 g, 1.34 mmol), **9b** was obtained as a white solid (70 % yield), mp = 122 °C; IR (KBr): 2977, 1728, 1451, 1285, 1246, 1150, 1081, 804, 757, 697 cm^-1^; ^1^H-NMR (DMSO-*d*_6_) δ 8.36 (s, 1H, H5); 8.15 (d, *J*_H7-8 _= 8.8 Hz, 1H, H7); 7.89-7.83 (m, 2H, H8, H3’); 7.58-7.55 (m, 2H, H2’, H6’); 7.46-7.44 (m, 1H, H5’); 7.29 (d, *J*_H2-3_ = 7.8 Hz, 1H, H2); 7.20 (d, *J*_H3-2_ = 7.8 Hz, 1H, H3); 3.47 (s, 3H, OCH_3_); 2.89 (s, 3H, CH_3_); 2.45 (s, 3H, CH_3_); 1.71 (s, 9H, 3CH_3_); MS (EI) *m/z* (%): 401 (M^+ •^, 1), 271 (100) (M^+ •^- OCH_3_ - CO_2_*t*Bu).

### 5-(5’,8’-Dimethyl-9’-tert-butoxycarbonyl-9’H-carbazol-3’-yl)-thiophene-2-carbaldehyde (**9c**)

Following a procedure identical to that described for **9a**, but using 5-bromo-2-thiophene-carboxaldehyde (**C**, 0.27 g, 1.54 mmol) and a reflux time of 24 h, compound **9c** was obtained as a yellow solid (90 % yield), mp = 168 °C; IR (KBr): 3428, 2971, 1739, 1657, 1428, 1225, 1149, 1061, 797, 665 cm^-1^; ^1^H-NMR (DMSO*-d*_6_) δ 9.90 (s, 1H, CHO); 8.38 (s, 1H, H4’) 8.07-8.05 (m, 2H, H1’, H3); 7.94 (d, *J*_2-1 _= 8.8 Hz, 1H, H2); 7.82 (d, *J*_H4-3 _= 3.9 Hz, 1H, H4); 7.24 (d, *J*_H7-6_ = 7.8 Hz, 1H, H7); 7.15 (d, *J*_H6-7_ = 7.8 Hz, 1H, H6); 2.81 (s, 3H, CH_3_); 2.40 (s, 3H, CH_3_); 1.66 (s, 9H, 3CH_3_); MS (ESI^+^): 405 (M^+^+1).

### 1,4-Dimethyl-6-pyridin-2-yl-9-tert-butoxycarbonyl-9H-carbazole (**9d**)

Following a procedure identical to that described for **9a**, but using 2-bromopyridine (**D**, 0.09 mL, 0.98 mmol), **9d** was obtained as a light brown solid (45 % yield), mp = 212 °C; IR (KBr): 3370, 2977, 1701, 1457, 1290, 1252, 1149, 1088, 803, 780, 670 cm^-1^; ^1^H-NMR (DMSO-*d*_6_) δ 8.66 (s, 1H, H5); 8.08 (d, *J*_H7-8 _= 8.8 Hz, 1H, H7); 8.07-7.95 (m, 1H, H6’); 7.75 (d, *J*_H8-7 _= 8.8 Hz, 1H, H8); 7.71-7.68 (m, 2H, H4’, H3’); 7.20-715 (m, 1H, H5’); 7.10 (d, *J*_H2-3_ = 7.8 Hz, 1H, H2); 7.02 (d, *J*_H3-2_ = 7.8 Hz, 1H, H3); 2.72 (s, 3H, CH_3_); 2.41 (s, 3H, CH_3_); 1.62 (s, 9H, 3CH_3_); MS (ESI^+^): 373 (M^+^+1).

### 5-(5’,8’-Dimethyl-9’-tert-butoxycarbonyl-9’H-carbazol-3’-yl)-furan-2-carbaldehyde (**9e**)

Following a procedure identical to that described for **9a**, but using 5-bromo-2-furaldehyde (**E**, 0.27 g, 1.54 mmol) and a reflux time of 24 h, compound **9e** was obtained as a yellow solid (60 % yield), mp = 110 °C; IR (KBr): 3430, 2975, 1744, 1672, 1450, 1285, 1149, 1084, 795 cm^-1^; ^1^H-NMR (DMSO*-d*_6_) δ 9.62 (s, 1H, CHO); 8.48 (s, 1H, H4’) 8.10 (d, *J*_H1’-2’ _= 8.8 Hz, 1H, H1’); 8.03 (d, *J*_H2’-1’ _= 8.79 Hz, 1H, H2’); 7.67 (d, *J*_H3-4 _= 3.9 Hz, 1H, H3); 7.34 (d, *J*_H4-3 _= 3.9 Hz, 1H, H4); 7.25 (d, *J*_H7-6_ = 7.8 Hz, 1H, H7); 7.16 (d, *J*_H6-7_ = 7.8 Hz, 1H, H6); 2.82 (s, 3H, CH_3_); 2.42 (s, 3H, CH_3_); 1.67 (s, 9H, *t*-Bu); MS (ESI^+^): 390 (M^+^+1).

### 1,1’,4,4’-Tetramethyl-9-tert-butoxycarbonyl-9H-dicarbazole (**9f**)

Following the identical procedure to that described for **9f**, but using 6-bromo-1,4-dimethyl-9-*tert*-butoxycarbonyl-9*H*-carbazole (**F**, 0.30 g, 0.80 mmol), **9f** was obtained as a white solid (40 % yield), mp = 214 °C; IR (KBr): 3439, 2979, 1726, 1453, 1295, 1248, 1154, 1083, 804, 541 cm^-1^; ^1^H-NMR (DMSO-*d_6_*) δ 8.40 (s, 2H, H5, H5’); 8.20-8.10 (m, 2H, H7, H7’); 8.00-7.90 (m, 2H, H8, H8’); 7.10-7.30 (m, 4H, H2, H3, H2’, H3’); 2.85 (s, 6H, 2CH_3_); 2.42 (s, 6H, 2CH_3_); 1.68 (s, 18H, 6CH_3_); MS (EI) *m/z* (%): 588 (M^+ •^, 1), 388 (100) (M^+ • ^- 2 CO_2_*t*Bu).

### General procedure for the deprotection of compounds **9a-f**

The preparation of 1,4-dimethyl-6-pyridin-3-yl-9*H*-carbazole (**5a**) is reported as a representative example: to a mixture of 1,4-dioxane (70 mL) and gaseous HCl (saturated solution) cooled to 0 °C compound **9a** (2.36 mmol) was added. The reaction mixture was heated to reflux for 48 h, then concentrated under reduced pressure. The oil formed residue was crystallized from acetonitrile. Compound **5a** was obtained as a yellow solid (95 % yield), mp > 260 °C; IR (KBr): 3426, 2518, 1470, 1258, 790, 671 cm^-1^; ^1^H-NMR (DMSO-*d_6_*) δ 11.51 (s, 1H, NH); 9.30 (d, *J*_H2’4’_ = 2.4 Hz, 1H, H2’); 8.84 (dd, *J*_H6’-5’ _= 4.4 Hz, *J*_H6’-4’ _= 1.2 Hz, 1H, H6’); 8.77 (part A of AB system, *J*_AB _= 8.3 Hz, *J*_HA-2’ _= 2.4 Hz, *J*_HA-6’ _= 1.2 Hz, 1H, H4’); 8.49 (s, 1H, H5); 8.03 (part B of AB system, *J*_AB _= 8.3 Hz, *J*_HB-6’ _= 4.4 Hz, 1H, H5’); 7.85 (d, *J*_H7-8 _= 8.8 Hz, 1H, H7); 7.68 (d, *J*_H8-7 _= 8.8 Hz, 1H, H8); 7.13 (d, *J*_H2-3_ = 6.8 Hz, 1H, H2); 6.91 (d, *J*_H3-2_ = 6.8 Hz, 1H, H3); 3.42 (1H, NH^+^Cl^-^); 2.85 (s, 3H, CH_3_); 2.52 (s, 3H, CH_3_); MS (ESI^+^): 273 (M^+^+1).

The following compounds were similarly obtained:

*6-(4’-Methoxyphenyl)-1,4-dimethyl-9H-carbazole *(**5b**). White solid (95 % yield), mp = 200 °C; IR (KBr): 3020, 1454, 1320, 1250, 1170, 1076, 799, 770, 676 cm^-1^; ^1^H-NMR (DMSO-d_6_) δ 10.00 (s, 1H, NH); 7.85 (s, 1H, H5); 7.58-7.56 (m, 2H, H2’, H6’); 7.40 (d, J_H8-7 _= 8.8 Hz, 1H, H8); 7.30 (d, J_H7-8 _= 8.8 Hz, 1H, H7); 7.05-7.00 (m, 2H, H3’, H5’); 6.98 (d, J_H2-3_ = 7.8 Hz, 1H, H2); 6.89 (d, J_H3-2_ = 7.8 Hz, 1H, H3); 3.85 (s, 3H, OCH_3_); 2.84 (s, 3H, CH_3_); 2.54 (s, 3H, CH_3_); MS (EI) m/z (%): 301 (M^+ •^, 100).

*5-(5’,8’-Dimethyl-9’H-carbazol-3’-yl)-thiophene-2-carbaldehyde *(**5c**). Yellow solid (90 % yield), mp = 262 °C; IR (KBr): 3435, 3267, 1634, 1430, 1241, 1058, 800, 669 cm^-1^; ^1^H-NMR (DMSO-d_6_) δ 11.58 (s, 1H, NH); 9.94 (s, 1H, CHO); 8,50 (s, 1H, H4); 8.11-8.10 (d, J_3’‑4’_= 3.9 Hz 1H, H3’); 7.89 (d, J_2-1_= 8.8 Hz 1H, H2); 7.83 (d, J_H4’-3’ _= 3.9 Hz 1H, H4’); 7.67 (d, J_1-2 _= 8.8 Hz 1H, H1); 7.19 (d, J_H7-6_ = 7.8 Hz, 1H, H7); 6.97 (d, J_H6-7_= 7.8 Hz, 1H, H6); 2.89 (s, 3H, CH_3_); 2.58 (s, 3H, CH_3_); MS (ESI^+^): 306 (M^+^+1).

*1,4-Dimethyl-6-pyridin-2yl-9H-carbazole *(**5d**). White solid (92 % yield), mp > 260 °C; IR (KBr): 3424, 2516, 1468, 1270, 800, 673 cm^-1^; ^1^H-NMR (DMSO-d_6_) δ 11.48 (s, 1H, NH); 8.64 (s, 1H, H5); 8.10 (d, J_H7-8 _= 8.8 Hz, 1H, H7); 8.09-7.92 (m, 1H, H6’); 7.79 (d, J_H8-7 _= 8.8 Hz, 1H, H8); 7.70-7.69 (m, 2H, H4’, H3’); 7.22-718 (m, 1H, H5’); 7.13 (d, J_H2-3_ = 7.8 Hz, 1H, H2); 7.07 (d, J_H3-2_ = 7.8 Hz, 1H, H3); 3.43 (1H, NH^+^Cl^-^); 2.73 (s, 3H, CH_3_); 2.45 (s, 3H, CH_3_); MS (ESI^+^): 273 (M^+^+1).

*1,1’,4,4’-Tetramethyl-9H-dicarbazole *(**5f**). White solid (94 % yield), mp = 254 °C; IR (KBr): 3454, 2987, 1457, 1300, 1254, 1187, 1067, 812, 570 cm^-1^; ^1^H-NMR (DMSO-d_6_) δ 10.20 (s, 2H, NH); 7.99-7.94 (m, 2H, H5, H5’); 7.59-7.38 (m, 4H, H7, H8, H7’, H8’); 7.15-6.98 (m, 4H, H2, H3, H2’, H3’); 2.85 (s, 6H, 2CH_3_); 2.55 (s, 6H, 2CH_3_); MS (EI) m/z: 388 (M^+ •^, 100).
